# Possible role of GABAergic depolarization in neocortical neurons in generating hyperexcitatory behaviors during emergence from sevoflurane anesthesia in the rat

**DOI:** 10.1042/AN20140004

**Published:** 2014-04-04

**Authors:** Byung-Gun Lim, Feng-Yan Shen, Young-Beom Kim, Woong Bin Kim, Yoon Sik Kim, Hee Chul Han, Mi-Kyoung Lee, Myoung-Hoon Kong, Yang In Kim

**Affiliations:** *Department of Anesthesiology and Pain Medicine, Korea University College of Medicine, Guro Hospital, 148 Gurodong-ro, Guro-gu, Seoul, Korea; †Department of Anesthesiology, Xinhua Hospital, Shanghai Jiaotong University School of Medicine, Shanghai 20092, China; ‡Department of Physiology and Neuroscience Research Institute, Korea University College of Medicine, 126-1 Anam-dong 5-ga, Seoul, Korea

**Keywords:** emergence agitation, GABA, KCC2, neocortex, NKCC1, sevoflurane, ACSF, artificial cerebrospinal fluid, AP-5, DL-2-amino-5-phosphonopentanoic acid, CNS, central nervous system, DF_GABA_, driving force for GABAergic response, DNQX, 6,7-dinitroquinoxaline-2,3-dione, E_GABA_, reversal potential of GABAergic response, GABA, γ-aminobutyric acid, I_muscimol_, muscimol-elicited current, KCC2, K^+^–Cl^−^ cotransporter isotype 2, LJP, liquid junction potential, MAC, minimum alveolar concentration, NKCC1, Na^+^–K^+^–2Cl^−^ cotransporter isotype 1, NMDA, *N-*methyl-D-aspartate, PAHB, post-anesthetic hyperexcitatory behavior, PCO_2_, partial pressure of carbon dioxide, PO_2_, partial pressure of oxygen, PSP, postsynaptic potential, RMP, resting membrane potential, Vpf, perforated patch potential

## Abstract

Hyperexcitatory behaviors occurring after sevoflurane anesthesia are of serious clinical concern, but the underlying mechanism is unknown. These behaviors may result from the potentiation by sevoflurane of GABAergic depolarization/excitation in neocortical neurons, cells implicated in the genesis of consciousness and arousal. The current study sought to provide evidence for this hypothesis with rats, the neocortical neurons of which are known to respond to GABA (γ-aminobutyric acid) with depolarization/excitation at early stages of development (i.e., until the second postnatal week) and with hyperpolarization/inhibition during adulthood. Employing behavioral tests and electrophysiological recordings in neocortical slice preparations, we found: (1) sevoflurane produced PAHBs (post-anesthetic hyperexcitatory behaviors) in postnatal day (P)1–15 rats, whereas it failed to elicit PAHBs in P16 or older rats; (2) GABAergic PSPs (postsynaptic potentials) were depolarizing/excitatory in the neocortical neurons of P5 and P10 rats, whereas mostly hyperpolarizing/inhibitory in the cells of adult rats; (3) at P14–15, <50% of rats had PAHBs and, in general, the cells of the animals with PAHBs exhibited strongly depolarizing GABAergic PSPs, whereas those without PAHBs showed hyperpolarizing or weakly depolarizing GABAergic PSPs; (4) bumetanide [inhibitor of the Cl^−^ importer NKCC (Na^+^–K^+^–2Cl^−^ cotransporter)] treatment at P5 suppressed PAHBs and depolarizing GABAergic responses; and (5) sevoflurane at 1% (i.e., concentration <1 minimum alveolar concentration) potentiated depolarizing GABAergic PSPs in the neurons of P5 and P10 rats and of P14–15 animals with PAHBs, evoking action potentials in ≥50% of these cells. On the basis of these results, we conclude that sevoflurane may produce PAHBs by potentiating GABAergic depolarization/excitation in neocortical neurons.

## INTRODUCTION

Emergence from sevoflurane anesthesia in preschool children is often complicated by the presence of hyperexcitatory behaviors, such as non-purposeful restlessness and agitation, disorientation, incoherence, and thrashing, which may cause serious injury to the patient, disruption or contamination of the surgical site, or removal of dressing, drains or intravenous catheters (Jerome, 1989; Olympio, 1991; Wells and Rasch, 1999). Despite such potential problems, it remains unknown how sevoflurane produces PAHBs (post-anesthetic hyperexcitatory behaviors).

Sevoflurane is a GABA_A_ receptor agonist and also a modulator, which potentiates GABA_A_ channel current (Wu et al., 1996). GABA (γ-aminobutyric acid) is well known to function as an inhibitory neurotransmitter in the mammalian CNS (central nervous system). However, it does not always elicit hyperpolarizing/inhibitory responses from postsynaptic neurons. In immature CNS neurons at fetal and neonatal stages of life and in some mature CNS neurons implicated in certain pathologies, such as epilepsy and neuropathic pain, GABA evokes depolarizing and even excitatory postsynaptic responses (Owens et al., 1996; Ben-Ari, 2002; Cohen et al., 2002; Coull et al., 2003; Baccei and Fitzgerald, 2004; Dzhala et al., 2005; Rheims et al., 2008) since the E_GABA_ (reversal potential of GABAergic response) in these cells is positive to the RMP (resting membrane potential) and/or the action potential threshold.

In patients manifesting PAHBs during emergence from sevoflurane anesthesia, the E_GABA_ values of the neurons in the brain regions involved in the generation of consciousness and arousal, such as the neocortex and thalamus (Newman, 1995; Schiff, 2008), may be positive to the RMP or action potential threshold because of the abnormality or delay in the maturing process of the mechanisms responsible for keeping the intracellular chloride concentration ([Cl^−^]_i_) low (He et al., 2014), such that GABA_A_ receptor-mediated transmission in these cells may be depolarizing or excitatory. Potentiation of this unusual GABAergic transmission by sevoflurane may be a key mechanism for the genesis of PAHBs. On the other hand, if GABA functions as an inhibitory transmitter in these neurons, the disinhibitory action of sevoflurane and its withdrawal effect on glutamatergic activity causing rebound excitation (similar to GABA withdrawal syndrome; Brailowsky et al, 1988) are potentially important mechanisms. In the current study, we sought the evidence for the first possibility. To this aim, we examined whether sevoflurane elicited PAHBs in immature rats, the neocortical neurons of which are known to respond to GABA with depolarization or excitation, whereas it failed to evoke PAHBs in mature rats in which hyperpolarization/inhibition replaces the depolarizing/excitatory GABAergic responses. Then, we examined whether 1% sevoflurane [i.e., concentration which is lower than 1 MAC (minimum alveolar concentration) (Franks and Lieb, 1996) and could be attained during emergence phase] potentiated depolarizing GABA_A_ receptor-mediated responses to elicit action potentials in neocortical neurons recorded in rat brain slices. Although the immature rats used in the current study are at developmental stages that correspond roughly to the third trimester of pregnancy and the very early postnatal life of full-term human babies (Workman et al., 2013), we thought that the young rats may make a good animal model in which observable robust effects shed light on the mechanisms that may take a dominating role in certain brain areas or subpopulations of neurons to generate PAHBs in preschool children.

## EXPERIMENTAL

### Animal care

Pregnant Sprague–Dawley rats purchased from Orient Bio were housed in a temperature-controlled (22–24°C) vivarium with a 12 h–12 h light–dark cycle. Male pups born from these rats and raised in this vivarium were used in the current study. The birth day was defined as P0 (postnatal day 0). The experimental procedures described below were approved by the Institutional Animal Care and Use Committee and conformed to the guidelines of the National Institutes of Health Guide for the Care and Use of Laboratory Animals. All possible efforts were made to minimize the number of animals used as well as their suffering.

### Anesthesia and scoring of PAHBs

Anesthesia on the rat was induced with 6% (v/v) sevoflurane gas over 1 min and then maintained with 3% sevoflurane gas for 9 min. These gases, which were generated with a sevoflurane and oxygen-fed, calibrated commercial vaporizer (Penlon Sigma Elite; Ohmeda, BOC Health Care), were supplied to the anesthesia chamber at a rate of 3 liters/min. In some cases, rats were anesthetized with propofol [15 mg/kg body weight, i.p. (intraperitoneal) injection], instead of sevoflurane, for comparison. The depth of anesthesia appeared not different between the two anesthetics, when tested by tail pinch. Following the cessation of the sevoflurane administration or after confirming deep propofol anesthesia, the rat was monitored for the possible appearance of PAHBs during the emergence phase. The duration of this phase (i.e., awakening time) was assumed to be the time until the return of the righting reflex and measured with a cut-off time of 30 min. For the detected PAHBs, two parameters were quantified. First, the total duration of the PAHB episodes was measured. Secondly, the most intense PAHB that the animal exhibited was scored with a scale that was a modification of the one used in a previous study to quantify seizure behaviors (Bough et al., 2002); i.e., it was given a score, ranging 0–6, based on its type [0–none; 1–trembling; 2–head bobbing or stereotypes; 3–unilateral forelimb or hindlimb clonus; 4–bilateral forelimb or hindlimb clonus; 5–side-to-side rolling; 6–wandering (i.e., scrambling along the monitoring chamber walls)], and a bonus score of 0, 0.5, or 1 on the basis of its sub-grade (0–mild; 0.5–moderate; 1–severe). The sum of the main and the bonus scores constituted the behavior score.

### Blood gas and pH measurement

To examine whether the PAHBs from sevoflurane anesthesia could be due to respiratory depression, we measured the PO_2_ (partial pressure of oxygen), the PCO_2_ (partial pressure of carbon dioxide), and pH of arterial blood using a blood gas analyzer (StatProfile® pHOx® Ultra, Nova Biomedical Corporation). Blood samples (0.2–0.25 ml) were obtained by means of percutaneous transcardiac puncture of the left ventricle immediately after the anesthetic administration (i.e., 1 min with 6% gas and 9 min with 3% gas). The ventricle was approached from the abdominal side through the diaphragm. The bright red color of the blood sample was regarded as indicating the accuracy of the cardiac puncture, the success rate of which was ~25%.

### Test of drug effects on the PAHBs

To investigate the possible involvement of NKCC (Na^+^–K^+^–2Cl^−^ cotransporter)-dependent, GABA_A_ channel currents in the genesis of the PAHBs following sevoflurane anesthesia, we examined the effects on the PAHBs of the NKCC inhibitor bumetanide (Payne et al., 2003). Bumetanide (5 μmole/kg body weight) was injected intraperitoneally 5 min before the sevoflurane anesthesia.

### Brain slice preparation

Brain slices were prepared in a manner similar to the one described previously (Kim et al., 2005). In brief, the brain was quickly excised from the rat anesthetized with sodium pentobarbitone (100 mg/kg body weight, i.p.; in the case of adult rats) or by ice-induced hypothermia (in the cases of newborn rats) and submerged in ice-cold ACSF (artificial cerebrospinal fluid, in mM: 124 NaCl, 1.3 MgSO_4_, 3 KCl, 1.25 NaH_2_PO_4_, 26 NaHCO_3_, 2.4 CaCl_2_, and 10 glucose). After being chilled for 1–2 min, the brain was trimmed to a block containing the frontal cortex. Coronal slices (350–450 μm) were cut from the tissue block in ice-cold ACSF, with the use of a vibroslicer (Campden Instruments). The slices were then transferred to a gas interface type recording chamber that was perfused with aerated [95% (v/v) O_2_/5% (v/v) CO_2_] ACSF (22–24°C), at a rate of 0.5–1 ml/min, by a peristaltic pump-driven or gravity-fed bath-perfusion system (Choi et al., 2008). Humidified 95% O_2_/5% CO_2_ gas was continuously blown over the slices to further ensure adequate oxygenation of cells in the tissues.

### Electrophysiological recording

Current- or voltage-clamp recordings were obtained from neocortical neurons (mostly regular-spiking cells in the layers 4–6) in the brain slices equilibrated for 1–8 h in the recording chamber. All of the electrophysiological recordings were performed with the use of the gramicidin-perforated patch-clamp technique, as described previously (Kim et al., 2011a). Voltage errors resulting from series resistance were compensated offline for voltage-clamp recordings and online for current-clamp recordings by using the bridge circuit. We corrected the LJP (liquid junction potential) prior to the experiments; we set the pipette potential to −8 mV just before the formation of patch configuration, knowing that the LJP was 15 mV (at 23°C), while the Vpf (perforated patch potential) arising from the gramicidin perforation was 7 mV. We assumed that the change in RMP detected when the recording mode was transformed from perforated to whole-cell configuration represented the Vpf. Signals from neurons amplified by Axoclamp-2B amplifier (bandwidth filter set at 10 kHz for current-clamp and 1 kHz for voltage-clamp recordings) were digitized and sampled at 50 μs intervals (Digidata 1320, pClamp 8.0; Molecular Devices).

### Electrical stimulation to evoke GABA_A_ receptor-mediated PSPs (postsynaptic potentials)

The GABA_A_ receptor-mediated PSPs were elicited by electrically stimulating a site near the recorded cell with a custom-made, bipolar tungsten electrode; constant current (0.1–0.5 mA) or voltage (5–20 V) pulses (biphasic square wave, 0.5-ms duration) were used for the stimulation.

### Drugs

All the drugs used in this study were from Sigma-Aldrich, except muscimol (Abcam), CGP 54626 hydrochloride (Tocris), and sevoflurane (Abbott). Muscimol (GABA_A_ receptor agonist) solution [for focal application to neurons with Y-tube method (Murase et al., 1989)] was prepared by dissolving this drug in ACSF, while bumetanide solution (for i.p. injection or application to brain slice) was made by diluting the stock solution (prepared with 0.025-N NaOH) with physiological saline or ACSF. The sevoflurane solution for bath-application was prepared by bubbling the gas mixtures of sevoflurane and 95% O_2_/5% CO_2_ generated by a calibrated commercial vaporizer (Penlon Sigma Elite; Ohmeda, BOC Health Care) in ACSF for >30 min. The concentrations of sevoflurane in the ACSF bubbled with 0.5 and 1% sevoflurane-containing gas mixtures, as determined by gas chromatography (Farthing et al., 1997), were 0.094 and 0.184 mM, respectively, values that were 92–94% of the theoretical ones. To minimize the loss of sevoflurane, we used high-quality polytetrafluorethylene tubings and valves. To record GABA_A_ receptor-mediated responses in isolation, we included AP-5 [DL-2-amino-5-phosphonopentanoic acid; NMDA (*N-*methyl-D-aspartate) receptor antagonist, 100 μM], DNQX (6,7-dinitroquinoxaline-2,3-dione; non-NMDA receptor antagonist, 20 μM), and CGP 54626 hydrochloride (GABA_B_ receptor antagonist, 1 μM) in the slice-perfusing medium.

### Statistical analysis

Numerical data are expressed as the mean±S.E.M. Student's *t* test and Mann–Whitney *U* test were used for the comparison of two independent datasets with and without normal distribution, respectively, whereas paired *t* test was performed for comparison of two dependent datasets. One-way ANOVA and pairwise comparison with Student–Newman–Keuls test were performed to compare multiple independent datasets with normal distributions, whereas Kruskal–Wallis one-way ANOVA on ranks were performed to compare datasets without normal distributions. Fisher Exact test was performed to see if there was a contingency between the two kinds of classification. *P*<0.05 was considered significant.

## RESULTS

### PAHBs occurring after sevoflurane anesthesia

**Figure 1:**
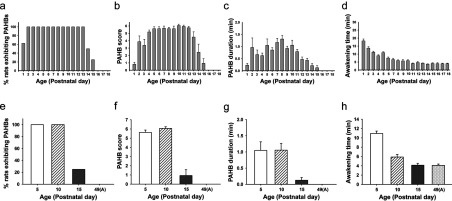
Plots against postnatal day of (1) the proportion of rats showing the PAHBs, (2) the score of PAHBs, (3) the total duration of the episodes of PAHBs and (4) the awakening time (i.e., duration of emergence phase) *a*–*d*, data from the same litter of rat pups (*n*=8) subjected to the sevoflurane anesthesia once per day between P1 and P18. *e–h*, data from different litters of rats subjected to the anesthesia on P5, P10, P15, and P49 (A) (i.e., at the adult stage) (*n*=8 each).

During emergence from sevoflurane anesthesia, P1–15 rats exhibited behaviors consisting of trembling, head bobbing, stereotypes, limb clonus, side-to-side rolling, and/or wandering. Usually, these PAHBs occurred in several episodes between 2 and 15 min after termination of anesthetic administration, each episode lasting a few to tens of seconds. The proportions of rats that exhibited the PAHBs on different postnatal days are graphically presented in Figure 1. Also, presented in this Figure are the scores of PAHBs, the total duration of the episodes of PAHBs and the awakening time (i.e., duration of emergence phase) on different postnatal days. The data in Figure 1, which were obtained from the same litter of rats tested on P1–18 (*a*–*d*) and from different litters of rats tested on P5, P10, P15, and P49 (*e*–*h*), demonstrate that the PAHBs occurred more frequently and robustly and lasted longer at P4–13 than other postnatal days. Furthermore, they show that the PAHBs decreased abruptly at around the end of the second postnatal week and were absent on P16 and thereafter.

To examine whether or not the PAHBs detected after sevoflurane anesthesia arose from respiratory depression caused by the anesthetic, we analyzed arterial blood for PO_2_, PCO_2_, and pH. In P10 rats (*n*=5), these parameters were all in normal range (PO_2_: 95±5 mmHg; PCO_2_: 35±2 mmHg; pH: 7.43±0.02). The same parameters were in normal range (PO_2_: 77±3 mmHg; pH: 7.37±0.01) in P5 rats (*n*=5) as well, with only the PCO_2_ (46±2 mmHg) suggesting a presence of mild hypercapnia. These results thus undermined the possible role of respiratory depression in the generation of the PAHBs.

Next, to test the possibility that the PAHBs following sevoflurane anesthesia are caused by sevoflurane at concentrations lower than 1 MAC that may occur during the elimination of sevoflurane after the cessation of anesthetic administration, we put P5 and P10 rats (*n*=6 each) in a chamber filled with 1% sevoflurane and monitored their movements. In ~5 min, each of these animals began to exhibit behaviors that were strikingly similar to those observed during emergence from sevoflurane anesthesia. Such behaviors continued to occur until the end of the monitoring period that lasted for 30 min. Thus, these results suggested that the PAHBs are caused by sevoflurane at concentrations lower than 1 MAC.

### PAHBs occur less frequently and less robustly in rats subjected to propofol, than sevoflurane, anesthesia

Several previous studies indicated that sevoflurane and propofol are drugs with higher and lower incidence of emergence agitation in humans, respectively (Nakayama et al., 2007; Bryan et al., 2009; Kanaya et al., 2014). Furthermore, a recent study suggested that emergence agitation in pediatric patients is lower in severity when anesthesia was maintained with propofol than sevoflurane (Pedersen et al., 2013). Therefore, we reasoned that, if the PAHBs observed in the current study are behaviors representing emergence agitation in humans, propofol anesthesia in the rat should produce PAHBs less frequently and less robustly than sevoflurane anesthesia. In the following experiment, we explored this possibility and found that propofol (15 mg/kg body weight, i.p. injection) produced PAHBs in a smaller proportion of rats than did sevoflurane (P5:10 of 14 *versus* 16 of 16, *P*=0.037; P10: 5 of 10 *versus* 16 of 16, *P*=0.004). Furthermore, we discovered that the PAHBs produced by propofol tended to be less robust than those elicited by sevoflurane (score: P5: 3.2±0.8, *n*=14 *versus* 5.5±0.2, *n*=16, *P*=0.101; P10: 1.5±0.6, *n*=10 *versus* 6.0±0.1, *n*=16, P≤0.001; duration: P5: 1.1±0.5 min, *n*=14 *versus* 1.1±0.2 min, *n*=16, *P*=0.019; P10: 0.1±0.0 min, *n*=10 *versus* 1.1±0.1 min, *n*=16, *P* ≤ 0.001). Thus, these results pointed to the possibility that the PAHBs in the rat represent emergence agitation in humans.

### The PAHBs occurring after sevoflurane anesthesia are suppressed by pre-emergent injection of the NKCC inhibitor bumetanide

**Figure 2:**
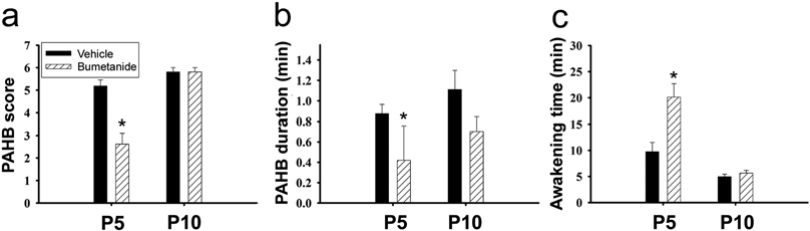
NKCC inhibition suppressed the PAHBs (*a–c*) Effects of bumetanide on the score of PAHBs, the total duration of the episodes of PAHBs and the awakening time in P5 and P10 rats (*n*=8 rats each). *, *P*<0.05.

In the immature rat brain, the high expression of NKCC1 (Na^+^–K^+^–2Cl^−^ cotransporter isotype 1; Cl^−^ importer) and the low expression of KCC2 (K^+^–Cl^−^ cotransporter isotype 2; Cl^−^ extruder) are known to underlie the production of depolarizing/excitatory GABAergic responses (Plotkin et al., 1997; Ben-Ari, 2002; Yamada et al., 2004). We thought that, if GABAergic depolarization/excitation plays a crucial role in the generation of PAHBs, inhibiting NKCC should result in the suppression of the PAHBs in immature rats. To test this possibility, we examined how the NKCC blocker bumetanide [5 μmole/kg body weight; i.p. injection (Dzhala et al., 2005; Edwards et al., 2010)] affected the PAHBs occurring in P5 and P10 rats. The control rats received vehicle injections. In P5 rats, the bumetanide injection 5 min prior to the sevoflurane anesthesia resulted in a significant decrease in the scores of PAHBs (vehicle: 5.2±0.3, *n*=8 versus bumetanide: 2.6±0.5, *n*=8, *P*=0.003; Figure 2a). In addition, the drug injection led to a significant reduction in the total duration of the episodes of PAHBs (0.9±0.1 to 0.4±0.3 min, *P*=0.01; Figure 2b) and to a significant increase in the awakening time (9.7±1.8 min to 20.1±2.6 min, *P*=0.007; Figure 2c). On the other hand, in P10 rats, the bumetanide injection had significant effects on neither of the behavioral score, the total duration of the episodes of PAHBs, and the awakening time (Figures 2a–2c). Thus, these results suggest that GABAergic depolarization/excitation plays a critical role in the production of PAHBs at P5, while they do not specify the exact site(s) of action of bumetanide.

### Sevoflurane excites neocortical neurons by potentiating inward GABA_A_ receptor-mediated current

**Figure 3:**
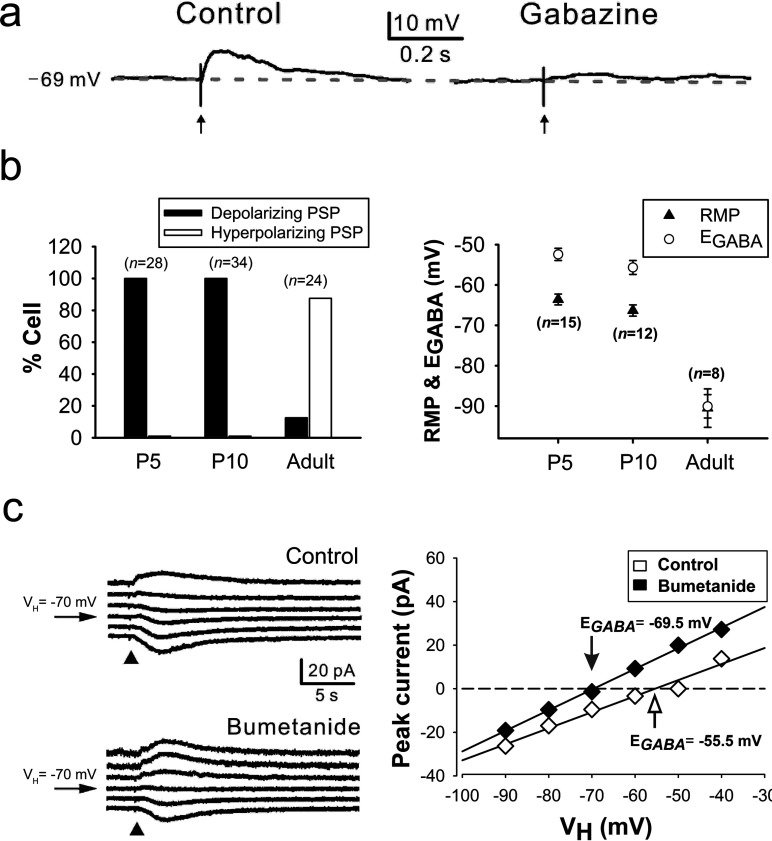
GABA_A_ receptor-mediated responses in neocortical neurons (**a**) Effect of gabazine (GABA_A_ receptor antagonist; 5 μM) on the depolarizing GABAergic PSP evoked in a neocortical neuron by electrical stimulation (↑) of a site near this cell. The PSP was evoked in the presence of AP-5 (NMDA receptor antagonist;100 μM), DNQX (non-NMDA receptor antagonist; 20 μM) and CGP 54626 hydrochloride (GABA_B_ receptor antagonist; 1 μM) in the slice-perfusing medium. (**b**) Proportions of neocortical cells showing depolarizing and hyperpolarizing GABA_A_ receptor-mediated PSPs at different developmental stages (left panel), and the E_GABA_ and RMP values of these neurons (right panel). (***c***) Effect of bumetanide on the E_GABA_ in a neocortical cell at P5. The E_GABA_ was estimated with the use of the currents (left panel) elicited at various holding potentials (V_H_) between −90 and −40 mV by focally applied muscimol (GABA_A_ receptor agonist; 10 μM, 10 ms; ▲) after blocking action currents with TTX (0.5 μM). The amplitudes of these currents were plotted against V_H_, and a linear regression was used to fit the data points (right panel). The intersection (arrow) of the regression line with the abscissa was taken as the E_GABA_.

**Figure 4:**
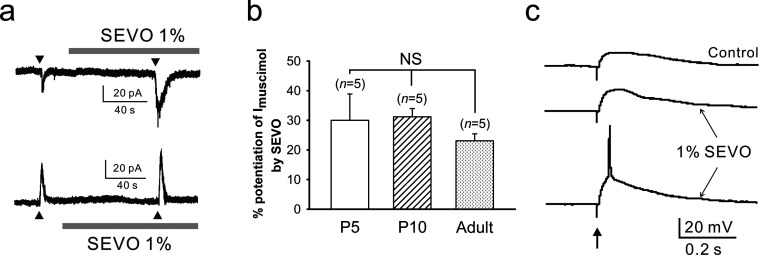
Effects of sevoflurane on GABAergic responses in neocortical neurons (**a**) Potentiation by sevoflurane (SEVO, 1%) of inward (upper panel) and outward currents (lower panel) elicited by focal muscimol application (10 μM, 10 ms; ▲) in neocortical neurons at P5 and adult stage, respectively. (**b**) Degree of the sevoflurane (1%) potentiation of muscimol (10 μM, 10 ms)-elicited current (I_muscimol_) in neocortical neurons at different developmental stages. NS: statistically insignificant. (**c**) Action potential triggering in a neocortical neuron at P5 by depolarizing GABAergic PSP potentiated by sevoflurane (SEVO, 1%). The bottom trace show the case where the potentiated PSP succeeded to evoke an action potential, whereas the middle one illustrates the occasion when the potentiated PSP failed to evoke spike. The depolarizing GABAergic PSP was evoked by electrical stimulation (↑) of a site near the recorded cell, in the presence of AP-5 (100 μM), DNQX (20 μM) and CGP 54626 hydrochloride (1 μM) in the slice-perfusing medium.

In the next set of electrophysiological experiments, we examined whether or not sevoflurane excited immature neocortical neurons by potentiating depolarizing GABA_A_ receptor-mediated PSPs occurring in these cells. In all of the neurons sampled in the neocortical slices of P5 (*n*=11) and P10 rats (*n*=12) (P5: 28 neurons; P10: 34 neurons), GABA_A_ receptor-mediated PSPs were depolarizing (Figures 3a and 3b, left panel), i.e., the E_GABA_ was positive to the RMP (Figure 3b, right panel), and in some cases, they were even excitatory (P5: 6 of 28 cells; P10: 6 of 34 cells), i.e., the E_GABA_ was positive not only to the RMP but also to the action potential threshold, which was −40 to −50 mV. On the other hand, in a majority of neocortical neurons (21 of 24 cells) of adult rats (*n*=5) studied for comparison, GABA_A_ receptor-mediated PSPs were hyperpolarizing/inhibitory (Figure 3b, left panel). The depolarizing GABA_A_ receptor-mediated PSPs occurring in the cells of P5, but not P10, rats were partially dependent on NKCC, as evidenced by the hyperpolarizing effect of bumetanide (10 μM) on the E_GABA_ (P5: −54.3±1.6 to −59.4±2.0 mV, *n*=7 cells; *P*<0.001; Figure 3c; P10: −51.9±1.0 to −52.9±0.9 mV, *n*=8; *P*=0.078). Sevoflurane at a concentration (1%) lower than 1 MAC increased I_muscimol_ [muscimol (GABA_A_ receptor agonist)-elicited current] regardless of its polarity in the neocortical cells of P5, P10, and adult rats (Figures 4a and 4b) and potentiated depolarizing GABAergic PSPs to elicit action potentials (Figure 4c) in 66.7 and 57.1% of the cells examined at P5 (*n*=9 neurons) and P10 (*n*=14 neurons), respectively. Notably, the sevoflurane-potentiated depolarizing GABAergic PSPs in most of these neurons were sub-threshold; i.e., their E_GABA_ values were negative to the action potential threshold in most of these cells. Nonetheless, they could elicit action potentials, presumably by evoking voltage-activated depolarizing events such as calcium potentials.

### In general, GABAergic PSPs are strongly depolarizing in the neocortical cells of P14–15 rats exhibiting PAHBs, whereas hyperpolarizing or weakly depolarizing in the cells of animals lacking PAHBs

**Figure 5:**
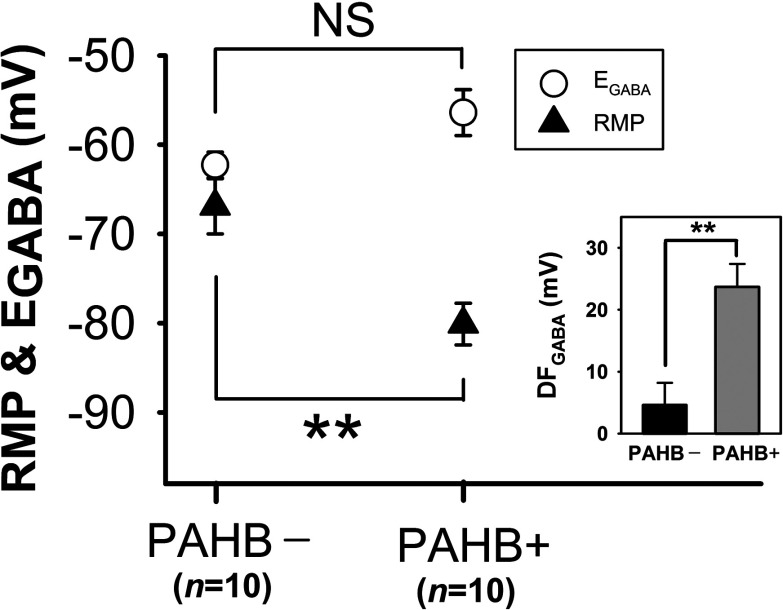
Comparison of P14–15 rats with and without PAHBs, in terms of the RMP and E_GABA_ in neocortical neurons PAHB+, neocortical neurons of rats having PAHBs; PAHB−, neocortical neurons of rats lacking PAHBs. Inset: DF_GABA_ (i.e., E_GABA_–RMP) values for PAHB+ and PAHB−. **, *P*<0.01; NS, statistically insignificant.

Taken together, the behavioral and electrophysiological results described above provide support for the notion that GABA_A_ receptor-mediated depolarization/excitation in neocortical neurons plays an important role in the genesis of PAHBs. In order to provide additional evidence for this hypothesis, we further investigated GABAergic transmission in neocortical neurons at P14–15, a period during which <50% of rats exhibit PAHBs (Figure 1). We reasoned that, if indeed GABAergic depolarization/excitation is crucial for the generation of PAHBs, the neocortical neurons of P14–15 rats with PAHBs should exhibit depolarizing/excitatory GABAergic PSPs, whereas the cells of P14–15 rats without PAHBs should not. Also, we thought that sevoflurane should potentiate these depolarizing/excitatory GABAergic PSPs, eliciting action potentials. Roughly consistent with these thoughts, the cells (*n*=20) of P14–15 rats (*n*=5) having PAHBs manifested moderately (*n*=4) to strongly (*n*=16) depolarizing GABAergic PSPs, as evidenced by the medium to large driving force, DF_GABA_ (driving force for GABAergic response) (i.e., E_GABA_–RMP) for them (23.7±3.7 mV, *n*=10; range: 9–49 mV), and sevoflurane (1%) potentiated the strongly depolarizing PSPs to give rise to action potentials (five of ten cells tested). On the other hand, the neurons (*n*=10) of P14–15 rats (*n*=3) lacking PAHBs exhibited either depolarizing (weak: *n*=4; moderate: *n*=3; strong: *n*=1) or hyperpolarizing (*n*=2) GABAergic PSPs, with the mean (±S.E.M.) DF_GABA_ being 4.6±3.6 mV (*n*=10; range: −15 to 20 mV). Interestingly, the difference in DF_GABA_ value between the two cell groups above (*P*=0.002) originated mainly from the difference in RMP (PAHB+: −80.1 ±2.3 mV *versus* PAHB−: −66.9±3.1 mV; *P=* 0.003), not E_GABA_ (PAHB+: −56.4±2.6 mV *versus* PAHB−: −62.3 ±1.5 mV; *P*=0.062) (Figure 5).

## DISCUSSION

In this study, combining behavioral and electrophysiological approaches, we sought the neural mechanism underlying the occurrence of sevoflurane PAHBs. We first found that the occurrence of PAHBs in the rat was well correlated with the presence of GABAergic depolarization/excitation in neocortical neurons; i.e., we discovered that: (1) PAHBs occurred in immature rats in which GABAergic effects on neocortical neurons are depolarizing or excitatory, but not in mature rats in which hyperpolarizing/inhibitory effects replace the depolarizing/excitatory ones, and (2) at P14–15 [i.e., the time point when excitatory-to-inhibitory switch in GABAergic neurotransmission in the neocortex is normally completed (Ben-Ari et al., 2007)], <50% of rats had PAHBs and, in general, the neocortical neurons of P14–15 rats with these behaviors exhibited strongly depolarizing GABAergic PSPs, whereas those without PAHBs manifested hyperpolarizing or weakly depolarizing GABAergic PSPs. Secondly, we discovered that bumetanide suppressed PAHBs and GABAergic depolarization/excitation in the neocortical neurons in P5, but not P10, rats. Thirdly and lastly, we found that sevoflurane at a concentration lower than 1 MAC potentiated depolarizing GABAergic PSPs in the neocortical neurons of P5 and P10 rats, and of P14–15 animals with PAHBs, evoking action potentials in ≥50% of these cells.

In the behavioral study, we noticed that some of PAHBs looked like seizures, and thus scored PAHBs using a scale that was a modification of the one used in an earlier study to quantify seizure behaviors (Bough et al., 2002). The PAHBs detected in this study, however, are unlikely to be seizures, in the light of that a previous study (Edwards et al., 2010), employing electroencephalogram recording, showed that emergence from 3-h sevoflurane anesthesia resulted in seizures in some P10–17 rats but not in P4–8 rats, whereas PAHBs occurred in not only all P2–13 rats subjected to 10-min sevoflurane anesthesia (see Figure 1), but also four of seven P5 and six of eight P10 rats anesthetized with sevoflurane for 3 h (B.-G. Lim, Y.-B. Kim, W.B. Kim, F.-Y. Shen and Y.I. Kim, unpublished work).

To assess the possible involvement of NKCC1-dependent, GABA_A_ receptor-mediated depolarization/excitation in the genesis of the sevoflurane PAHBs, we took the approach of examining the effect on the PAHBs of bumetanide administered before sevoflurane anesthesia. While systemic administration of bumetanide has been used in earlier studies to inhibit neuronal Cl^−^ uptake via NKCC1 and to thereby reduce depolarizing actions of GABA (Edwards et al., 2010; Dzhala et al., 2005), it was found more recently that brain tissue levels of bumetanide remain negligible compared to plasma levels (Brandt et al., 2010; Cleary et al., 2013). Considering the dose, route, and timing of administration of bumetanide (5 μmol/kg body weight, i.p.; 5 min before sevoflurane anesthesia), it is possible that the effects of bumetanide on the PAHBs were not mediated by inhibition of NKCC1 in neocortical neurons. However, our estimatation of the bumetanide level in the brain [which was based on the results of the recent studies of Brandt et al. (2010) and Cleary et al. (2013)] indicates that the brain tissue concentrations of bumetanide for the period of time during which PAHBs are monitored (0.027–0.2 μM) are comparable with the inhibition constant (K_i_) of this drug (~0.1 μM) (Russell, 2000), and thus, that bumetanide exerted its effect against the PAHBs by blocking NKCC1.

Taken together, the results of the present study support the notion that the excitation of neocortical neurons by sevoflurane through the potentiation of GABAergic depolarization plays an important role in the genesis of PAHBs in immature rats. In addition, our results suggest that NKCC1 contributes to the occurrence of the GABAergic depolarization/excitation in P5, although not in P10, rats.

In pediatric human patients, a variety of neurotransmitter/modulator systems are thought to be involved in the etiology of sevoflurane emergence agitation since ketamine, fentanyl, α2-agonists, etc. are effective in reducing or preventing this undesirable behavior (Dahmani et al., 2010). In the human being, GABAergic depolarization/excitation also may be involved in the production of sevoflurane emergence agitation, as in immature rats. In humans, however, the excitatory-to-inhibitory switch in GABAergic neurotransmission is known to occur well before term birth and sevoflurane emergence agitation is mostly a problem at 2–5 years of age (Aono et al., 1997; Vlajkovic and Sindjelic, 2007)–a period during which GABAergic neurotransmission is assumed to be of inhibitory nature (Xu et al., 2011). Furthermore, several human studies have shown that the pre-emergent injection of GABA agonists, such as midazolam or propofol, lowers (rather than raises) the incidence of emergence agitation in children subjected to sevoflurane anesthesia (Ko et al., 2001; Aouad et al., 2007; Kim et al., 2011b), thus arguing against the possibility that GABAergic depolarization/excitation plays an important role in the etiology of sevoflurane emergence agitation. Nevertheless, we cannot rule out this possibility since other human studies (Viitanen et al., 1999; Breschan et al., 2007) and a meta-analysis (Dahmani et al., 2010) have reported the lack of effect of midazolam against the emergence agitation and, more importantly, it has not been determined whether GABA is purely inhibitory in the neocortical neurons of children at 2–5 years of age.

The exact nature of GABA effect in these cells is not well documented. The transition between the excitatory and inhibitory modalities of GABAergic neurotransmission may not be completed before birth, contrary to popular belief, and it may be quite variable in timing across individuals. Consistent with this line of thought, a study of Dzhala et al. (2005) performed with the use of human parietal lobe autopsy specimens has demonstrated that the developmentally regulated down-regulation of NKCC1 and up-regulation of KCC2 in neocortical neurons are still in progress during the first year of life such that high [Cl^−^]_i_ results, which in turn could contribute to GABAergic excitation. In addition, several studies have shown that, in normal mature rats, GABA exerts a depolarizing or even excitatory effect in certain CNS neurons (DeFazio et al., 2002; Szabadics et al, 2006; Nakane and Oka, 2010; Haam et al., 2012). In 2–5-year-old children exhibiting sevoflurane emergence agitation, GABAergic transmission in neocortical neurons could be depolarizing/excitatory because NKCC1down-regulation and/or KCC2 up-regulation are delayed in these cells. Alternatively, GABA could be depolarizing because the RMP is negative to the E_GABA_, as in the neocortical neurons of P14–15 rats with PAHBs (Figure 5). Another potential reason that GABA could be depolarizing/excitatory in the neocortical neurons of 2–5-year-old children with the emergence agitation may be certain physiological need or pathological condition; chronic hyperosmotic stress or epileptic and neuropathic conditions have been reported to cause depolarizing shift of E_GABA_ in related CNS neurons producing GABAergic excitation (Cohen et al., 2002; Coull et al., 2003; Kim et al., 2011a; Kim et al., 2013). Whether or not GABAergic depolarization/excitation indeed occurs in neurons implicated in the generation of consciousness and arousal such as neocortical cells and plays a key role in the genesis of sevoflurane emergence agitation in humans remains to be determined. A promising approach toward testing this hypothesis could be to first assess the possible prophylactic effect of bumetanide against the emergence agitation particularly in younger children.
